# Downstream Processing of Itraconazole:HPMCAS Amorphous Solid Dispersion: From Hot-Melt Extrudate to Tablet Using a Quality by Design Approach

**DOI:** 10.3390/pharmaceutics14071429

**Published:** 2022-07-07

**Authors:** Saurabh M Mishra, Margarethe Richter, Luis Mejia, Andreas Sauer

**Affiliations:** 1 SE Tylose USA Inc., Pharmaceutical Application Laboratory, Totowa, NJ 07512, USA; saurabh.mishra@setyloseusa.com (S.M.M.); luis.mejia@setyloseusa.com (L.M.); 2 Thermo Fisher Scientific, Pfannkuchstrasse 10-12, 76185 Karlsruhe, Germany; margarethe.richter@thermofisher.com; 3 SE Tylose GmbH & Co. KG, Kasteler Str. 45, 65203 Wiesbaden, Germany

**Keywords:** amorphous solid dispersion (ASD), hypromellose acetate succinate, hot-melt extrusion (HME), downstream processing, milling, tablet compaction, scale-up

## Abstract

The downstream processing of hot-melt extruded amorphous solid dispersions (ASDs) into tablets is challenging due to the low tabletability of milled ASDs. Typically, the extrudate strand is sized before milling, as the strand cannot be fed directly into the milling system. At the lab scale, the strand can be sized by hand-cutting before milling. For scaling up, pelletizers or chill roll and flaker systems can be used to break strands. Due to the different techniques used, differences in milling and tablet compaction are to be expected. We present a systematic study of the milling and tableting of an extruded ASD of itraconazole with hypromellose acetate succinate (HPMCAS) as a carrier polymer. The strand was sized using different techniques at the end of the extruder barrel (hand-cutting, pelletizer, or chill roll and flaker) before being milled at varying milling speeds with varying screen sizes. The effects of these variables (sizing technology, milling speed, and screen size) on the critical quality attributes (CQAs) of the milled ASD, such as yield, mean particle size (D50), tablet compaction characteristics, and tablet dissolution, were established using response surface methodology. It was found that the CQAs varied according to sizing technology, with chill roll flakes showing the highest percentage yield, the lowest D50, and the highest tabletability and dissolution rate for itraconazole. Pearson correlation coefficient tests indicated D50 as the most important CQA related to tabletability and dissolution. For certain milling conditions, the milling of hand-cut filaments results in similar particle size distributions (PSDs) to the milling of pellets or chill roll flakes.

## 1. Introduction

The poor aqueous solubility of drug molecules remains the most prominent challenge in pharmaceutics, with as much as 70% of drug molecules showing low aqueous solubility in drug development stage. Since the oral absorption of drugs depends on their solubilization and permeation, the low solubility of drugs causes lower oral absorption and erratic bioavailability of drugs [1,2]. To address this, various approaches have been applied to enhance the solubility and bioavailability of drugs. Among these approaches, the preparation of amorphous solid dispersions (ASDs) from crystalline drugs in the presence of an amorphous carrier polymer is one of the most popular methods [3,4]. Thermodynamically, ASDs remain in a metastable state, having significantly higher free energy than crystalline states, thus showing higher aqueous solubilities, while, kinetically, due to strong interactions between drugs and polymers, nucleation can be inhibited or delayed, thus maintaining supersaturation of a drug over a long period of time in dissolution media [2,5]. This interplay of thermodynamic instability and kinetic stabilization makes ASD one of the most suitable formulation strategies to enhance the solubility of drugs. For commercial-scale manufacturing of ASDs, spray drying (SD) based on solvent evaporation and hot-melt extrusion (HME) based on fusion/melting are the most commonly employed methods [6]. Despite the considerable interest in ASD formulations, downstream processing studies for the development of ASD tablets are limited due to various challenges associated with the properties of ASDs [7]. In our previous study, we reported on the downstream processing of a spray-dried ASD of nifedipine using hypromellose acetate succinate (HPMCAS) as a carrier polymer [8]. However, the challenges faced in the downstream processing of ASDs prepared by spray drying are totally different from those encountered in the production of HME-based ASDs. For instance, spray-dried ASD particles are small and hollow in structure, showing lower bulk density and poor powder flow [7,8]. Thus, densification by dry granulation using roller compaction is usually applied as a downstream process to improve the particle size and flow of spray-dried ASD powders. In the case of HME-based ASDs, sizing and milling of extrudates usually produces materials with high bulk densities and good flowability, rendering granulation prior to tableting unnecessary [7]. However, the milling and tableting of ASD extrudates is challenging due to the nature of the HME process involving the melting and solidification of a drug–polymer mixture, which causes the high viscoelasticity and reduces the brittleness of extrudates [7,9].

HME of drugs and polymers involves several steps, including the conveying of materials inside a barrel, heat-up and plasticization, the melting and kneading and mixing of materials, degassing, pushing of the melt through a die, and further sizing/cooling of the strand into the desired shape for milling [10,11]. Thus, depending on the die or the equipment used, different types of ASD feedstocks for milling can be achieved. At the laboratory scale, melt extrudates in the form of filaments of different diameters and lengths are commonly used as feedstocks for milling. Milled ASD can be converted into tablets or capsules [12]. Extruded filaments can also be pelletized, or the molten mixture can be pumped through the die, cooled, and formed into a pellet with a definite shape and size which can either be milled for further size reduction or directly put into capsule form [13]. Another approach involves passing the molten extrudates through chill rolls, after which, once cooled, the resultant sheet is crushed by a flaker and collected to be milled for subsequent compression into tablets [14]. Thus, the sizing of extruded strands and milling remain the most important downstream processes in the formulation development of ASD tablets from hot-melt extrudates. The importance of milling and resultant particle size reduction (PSD) in the formulation development of solid dosage forms has been well established. For example, the effect of PSD on tablet tensile strength, due to its large influence on bonding area and bonding strength, has been reported by Chang and Sun [15]. Similarly, the significant influence of PSD on the dissolution rates of APIs, due to its proportional effects on the surface areas of APIs, has been well established in the literature [16,17]. However, a careful review of various reports in the literature on ASD formulations using HME suggests that, although the milling of extrudates has been reported, a robust platform for downstream processing, including sizing, milling, and tableting, is limited. For example, Agarwal et al. [12] reported HME of compound X with three different amorphous polymers to prepare highly soluble ASD tablets. A two-step process was carried out to first break the long extruded strand into pieces followed by further milling to achieve granules for tablet compression [12]. In another study which sought to prepare an ASD of celecoxib by HME, Grymonpré et al. [18] reported using a knife mill for the milling of ASD extrudates followed by passage through a sieve (no. 150) in the development of a tablet formulation. Similarly, Davis et al. [19] and Solanki et al. [20] reported the successful formation of an Itraconazole ASD using HME; however, before tablet compression, extrudates were milled using either a ball mill [19] or lab-scale cryomilling [20], respectively. In both cases, the milled extrudates were passed through a sieve to achieve the desired particle size. Hence, as can be seen in the summarized literature, downstream processing is mostly carried out by cold-cutting extrudates and/or milling followed by sieving to achieve the desired particle size for the further formulation development of tablets. As these processes are not sufficiently robust and are time-consuming due to the additional step of sieving after milling, establishing a design strategy for producing products of consistently high quality is a challenge. Moreover, the aforementioned reported methodologies were developed in small-scale studies for application at the laboratory level and information is lacking about the transition to pilot or production scale.

**Figure 1:**
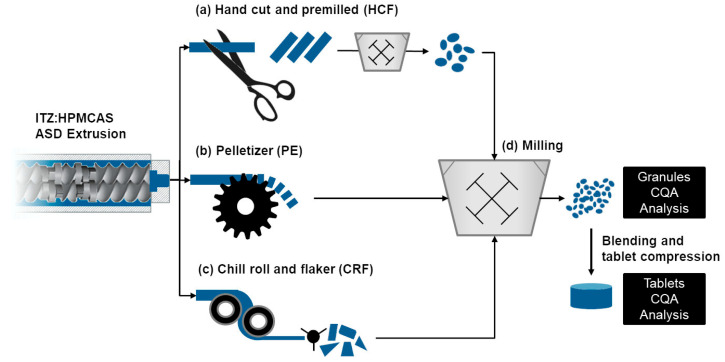
Downstream processing of extruded strands by hand-cutting (**a**), pelletizing (**b**), and chill roll technology (**c**) and subsequent milling (**d**).

Thus, in the present study an attempt has been made to develop a robust downstream processing platform, including the sizing, milling, and tableting of ASD strands prepared by HME. Itraconazole was selected as a poorly soluble API model and a medium substitution and particle size grade of hypromellose acetate succinate (HPMCAS AS-MMP) was used as a carrier polymer to prepare the ASD. Itraconazole (ITZ) is an antifungal agent that can be administered both orally and intravenously. However, the oral bioavailability of ITZ is poor due to extremely low intrinsic solubility (~4 ng/mL) and a pKa value of 3.7 [21]. Different approaches have been used to enhance the solubility of ITZ; among them, the preparation of an ASD is the most popular. At present, there are three marketed formulations of Itraconazole (Onmel^®^, Sporanox^®^, and Tolsura^®^) which use ASD approaches [6]. Among them, except Onmel^®^, Sporanox^®^, and Tolsura^®^ are available in capsule dosage form [6]. The application of HPMCAS for enhancing the solubility of ITZ in a hot-melt extrusion process has been reported in detail by Solanki et al. [20,21] and others [22,23]. In the present study, a blend of ITZ and HPMCAS in a ratio of 20:80 was hot-melt extruded and subjected to three different sizing technologies before being milled into granules suitable for tablet compression (Figure 1).

The milling feedstock was prepared either by sizing the extruded strand by hand-cutting (a), pelletization (b) or a chill roll and flaker process (c). All three ASD feedstocks were milled at varying milling speeds and with varying sieve sizes in a hammer mill (d) and the effect of sizing technology (a–c) and milling conditions (d) on the CQAs of the ASD, such as percentage yield after milling, granule particle size, tablet tensile strength, and release of ITZ from the ASD tablets, were analyzed using a Quality by Design (QbD) approach. Further correlations between different CQAs of the ASD were established using Pearson correlation coefficient tests (r). In summary, we report a comprehensive study of the manufacture of an ITZ ASD with HPMCAS as a carrier polymer by hot-melt extrusion. A QbD approach with three different sizing technologies before milling and further downstream processing has been carried out which, to the best of our knowledge, has not been reported before.

## 2. Materials and Methods

### 2.1. Materials

Itraconazole (ITZ) was purchased from Ash Ingredients (Glen Rock, NJ, USA). Hypromellose acetate succinate (HPMCAS, Shin-Etsu AQOAT^®^ AS-MMP) and low-substituted hydroxypropyl cellulose (L-HPC NBD-021) were received from the Shin-Etsu Chemical Co., Ltd. (Tokyo, Japan). MCC PH 102 was received from IFF (Wilmington, DE, USA). Silicon dioxide (Aerosil^®^ 200) was purchased from the Evonik Corporation (Parsippany, NJ, USA). All other reagents were of analytical grade and were used as received.

### 2.2. Methods

#### 2.2.1. Preparation of Milling Feedstock

The feed rate of materials, screw speed, and the die size of the extruder were varied according to the sizing process.

**Figure 2:**
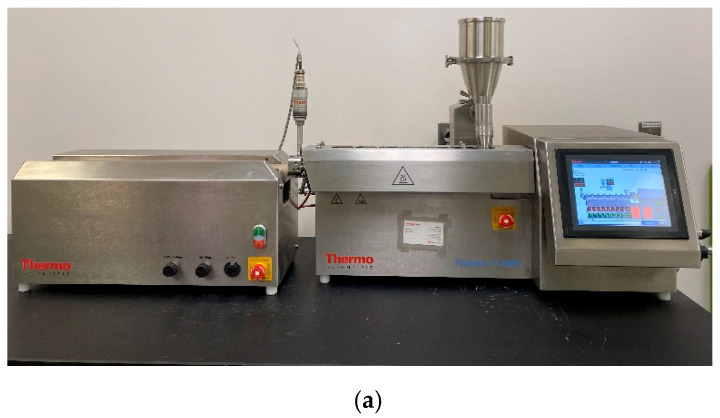

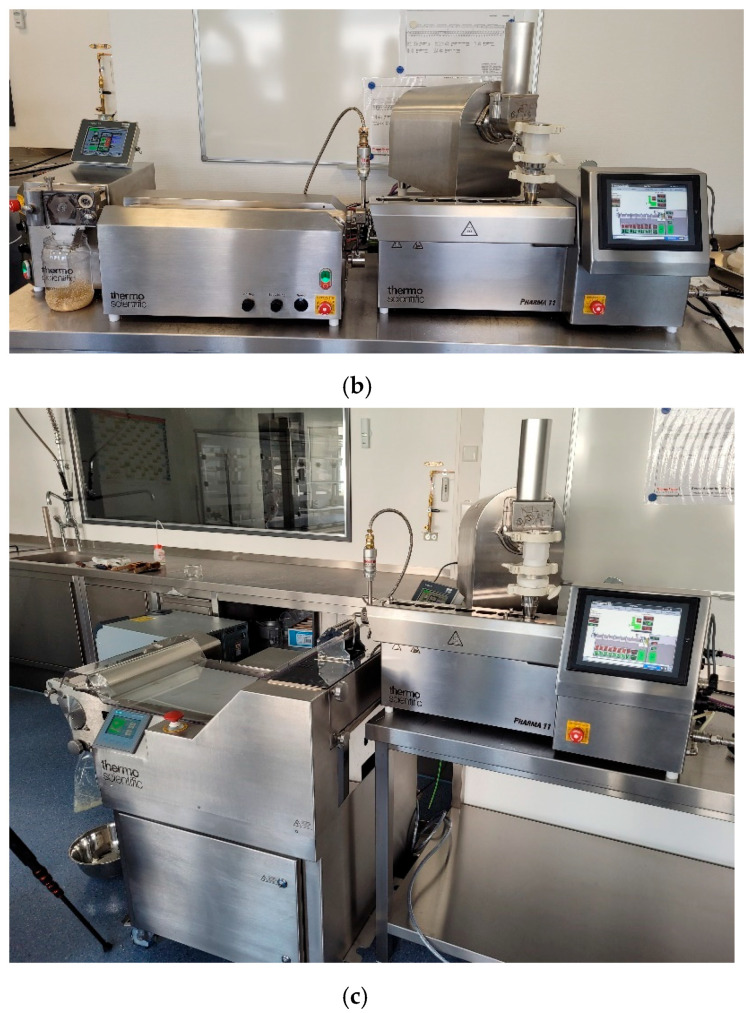
(**a**). Photograph of the manufacturing set-up for the HCF feedstock. (**b**). Photograph of the manufacturing set-up for the PE feedstock. (**c**). Photograph of the manufacturing set-up for the CRF feedstock.

(a) Hand-cut filaments (HCFs): A blend of ITZ:AS-MMP (20:80) was fed through a gravimetric feeder at 2.5 g/min into the extruder barrel (Pharma 11, Thermo Scientific, Karlsruhe, Germany; 165 °C, 2 mm die, 150 rpm) (Figure 2a). The screw configuration is presented in the Supplementary Materials (Figure S1). The extruded strand was cooled on a conveyor belt and cut into 5–8 cm filaments using a knife. The filaments were premilled using a hammer mill (Fredrive lab, Frewitt, Hillsborough, NJ, USA; 15,000 rpm, 4 mm screen). The premilled filaments were collected and used as premilled hand-cut feedstock (HCF) for further milling studies (Figure 1).

(b) Pellets (PEs): A blend of ITZ:AS-MMP (20:80) was extruded at a feed rate of 8.3 g/min (Pharma 11, Thermo Scientific, Karlsruhe, Germany; 165 °C, 2 mm die, 400 rpm) using the same screw configuration as for the HCF feedstock (Figure S1). The extrudate was placed on an air-cooled conveyor and introduced into the pelletizer (Varicut, Thermo Scientific, Karlsruhe, Germany; pelletizer speed 2, cutting speed L3) (Figure 2b). The pellets were collected and used as pellet (PE) feedstock for further milling studies (Figure 1).

(c) Chill roll flakes (CRFs): A blend of ITZ:AS-MMP (20:80) was extruded at a feed rate of 8.3 g/min (Pharma 11, Thermo Scientific, Karlsruhe, Germany; 165 °C, 6 mm die, 400 rpm) using the same screw configuration (Figure S1) as for the HCF feedstock. The strand was fed into a chill roll (Thermo Scientific^TM^ Pharma 16 Chill Roll, Thermo Scientific, Karlsruhe, Germany; gap 300 µm, cooling water 30 °C). The resulting film sheet was then granulated by a flaker (Figure 2c). The flakes were collected and used as chill roll flake (CRF) feedstock for further studies (Figure 1).

#### 2.2.2. Design of Experiments

A DoE was set up to ascertain the effect of feedstock type used and milling parameters on milled ASD CQAs and tablet CQAs using response surface methodology. Type of feedstock used, milling speed (rpm), and mesh screen size (mm) were selected as independent variables (Table 1). The independent factors were categorized into three levels: −1, 0, and +1 (low, medium, and high, respectively), and a total of 18 experiments was generated, including two center points (Table 2). Percentage yield, mean particle size D50 (µm), tablet tensile strength (MPa), and ITZ release (μg/mL) from compacted tablets at 30 min (Q30) and 60 min (Q60) were selected as the CQAs of ITZ-HPMCAS ASD (Table 1). The relationship between the independent variables and the CQAs of ITZ ASDs was quantified by a second-order polynomial equation (Equation (1)) [24]:

Y = B_0_ + B_1_X_1_ + B_2_X_2_ + B_3_X_3_ + B_12_X_1_X_2_ + B_23_X_2_X_3_ + B_13_X_1_X_3_ + B_22_X_2_^2^ + B_33_X_3_^2^ (1)

where Y is predicted or measured response; B_0_ is model constant or intercept; X_1_, X_2_, and X_3_ are independent variables—type of feedstock used, milling speed, and mesh diameter respectively. B_1_, B_2_, and B_3_ are linear coefficients, while B_12_, B_23_, and B_13_ are interaction terms or cross-product coefficients, and B_22_ and B_33_ are quadratic coefficients. The DoE was constructed using commercially available software (JMP 12, SAS Institute Inc., Cary, NC, USA) and the effect of each independent variable on the response variables was analyzed using multiple regression analysis and ANOVA with a 95% confidence interval (*p* < 0.05). As type of feedstock used (X_1_) was a categorical variable, effect plots were generated to demonstrate the effect of each feedstock on the CQAs of milled extrudates. An effect plot displays a change in response when a factor varies from one level to another with all other factors kept constant at their average values [25]. The effects of milling speed (X_2_) and mesh size (X_3_) were illustrated using response surface plots with regions of maxima and minima [24]. As some CQAs were expected to be interdependent, to establish the relationships between the CQAs of extrudates, Pearson correlation coefficient tests (r) were used. Based on the relationships, values of −1 and +1 were assigned, indicating negative and positive correlations, respectively [24]. In total, 18 milling experiments were conducted (Table 2).

#### 2.2.3. Compression of Milled Extrudates into Tablets

Milled ASD extrudates from all 18 experiments were blended according to Table 3. Preliminary trials using HCF feedstock (Table S1 and Figure S2) revealed that 50% loading of milled ASD gives tablets with sufficient tensile strength for industrial processing (>1.7 MPa) [26]. The blend was compacted into tablets (11.5 mm curved face punch, 650 mg tablet weight, 65 mg of ITZ, 200 MPa compaction pressure) using a single punch press (HANDTAP-200, Ichihashi Seiki, Kyoto, Japan).

#### 2.2.4. Percentage Yield after Milling

One-hundred grams of feedstock was milled for 90 s, according to Table 2, and percentage yield was calculated using Equation (2):

percentage yield = (w_0_ − w_1_)/w_0_ × 100 (2)

where w_0_ is the weight of feedstock (g) and w_1_ is the weight of outputted material after the milling process (g).

#### 2.2.5. Particle Size Distribution after Milling

Mean particle size, D50 (μm) of milled ASD extrudates according to the experimental design (Table 2), was determined using a laser diffraction system composed of Helos (H1740) and Rodos R6 (Sympatec GmbH, Clausthal-Zellerfeld, Germany) devices with a dispersion pressure of 1 bar.

#### 2.2.6. Carr’s Compressibility Index and Hausner Ratio 

From the mass and volume of the powder, the bulk density of the powder material was computed. Tapped density was calculated from the mass and the tapped volume of the powder using a Caleva Tap Density Tester (Type TD2, Dorset, UK). Carr’s compressibility index and Hausner ratio were calculated using bulk and tapped density, and flow properties were classified according to USP guidelines [28,29,30].

#### 2.2.7. DSC Analysis of Milled Extrudates

DSC analysis of each milled feedstock was carried out using a PerkinElmer DSC 6000 with a heat–cool–heat cycle. Accurately weighed 5–6 mg samples were crimped into an aluminum lid and heated in a temperature range of 25–200 °C at a heating rate of 2.5 K/min and cooled to 25 °C at 10 K/min, then heated again to 200 °C at a rate of 2.5 K/min. The glass transition temperature (T_g_) and melting endotherm of ITZ were determined using PyrisTM Manager software (PerkinElmer, Shelton, CT, USA).

#### 2.2.8. pXRD of Milled Extrudates

For pXRD analysis, a Thermo Scientific ARL Equinox 100 powder diffractometer (X-ray micro source Cu K_α_ radiation; beam size: 5 mm × 50 µm; 40 kV, 0.9 mA) equipped with a CPS 180 detector was used to measure diffraction patterns (reflection mode; ω = 4°) up to 110°2Θ. Thermo Scientific SolstiX XRD software was used for data acquisition and handling. 

#### 2.2.9. SEM Analysis of Milling Feedstock

For microscopical analysis of feedstocks, Scanning Electronic Microscopy (SEM) TM3000 (Hitachi, Tokyo, Japan) was used (15 kV, analysis mode).

#### 2.2.10. Solid Fraction

The true density of milled extrudates (for experiments 4, 5, 9, 11, 15, 16, as per Table 2), was measured by helium pycnometry (UltraPyc 5000, Anton Paar, Ashland, OH, USA). The true density of L-HPC NBD-021, MCC 102, magnesium stearate, and silicon dioxide were measured by helium pycnometry (AccuPyc II 1340, Micromeritics Instruments Corp., Norcross, GA, USA). Solid fraction of tablet was calculated as per Mishra and Rohera [31].

#### 2.2.11. Tablet Tensile Strength

Hardness of tablets and dimensions (thickness and diameter) were determined using a hardness tester (TB125, Erweka GmbH, Langen, Germany) (n = 6). The tensile strength of tablets (σ) was calculated according to Equation (3) for curved face tablets [32]:

σ = 10F/ (πD^2^ (2.84t/D − 0.126t/W + 3.15W/D + 0.01) (3)

where F is the breaking force of tablets (N), D and t are the diameter (mm) and thickness (mm) of tablets, and W is the wall height (mm) of round biconvex tablets, respectively.

#### 2.2.12. Compaction Analysis

For compaction analysis, feedstocks (HCF, PE, and CRF) milled at two different milling speeds (5000 and 15,000 rpm) using the same mesh size (1 mm) (experiments 4, 5, 9, 11, 15, 16, as per Table 2) were compacted into tablets (11.5 mm curved face punch, 650 mg tablet weight, 65 mg of ITZ, 25–250 MPa compression pressure) using a single punch press (HANDTAP-200, Ichihashi Seiki, Kyoto, Japan). The tabletability profile was determined by plotting tensile strength vs. compression pressure, which indicates the consolidation of particles with the application of compression pressure, while compressibility and compactibility indicate the degrees of volume reduction in the powder bed and consolidation of particles, respectively. The compressibility of ASD formulation was determined by plotting solid fraction vs. compression pressure, and compactibility was determined by plotting tensile strength vs. solid fraction of tablet [33].

#### 2.2.13. Tablet Disintegration Time

Disintegration time of the ASD-loaded tablet was analyzed using a disintegration apparatus (ZT72, Erweka GmbH, Langen, Germany) as per USP specifications. The disintegration test was carried out using 900 mL of purified water maintained at 37 ± 0.5 °C. Each determination was carried out one tablet at a time for six tablets. Means ± SDs are reported (n = 6).

#### 2.2.14. Tablet Dissolution Profile

The in vitro drug release studies of ASD tablets containing 65 mg of ITZ per tablet were conducted using a USP dissolution apparatus 2 (Distek 2500, Distek Inc., North Brunswick, NJ, USA). Since HPMCAS AS-MMP is an enteric polymer and only dissolves at pH > 6, the dissolution studies of ASD tablets were performed in 1000 mL of phosphate-buffered solution (pH 6.8) maintained at 37 ± 0.5 °C, with a stirring speed of 75 rpm maintaining sink condition [34,35]. Five-milliliter dissolution samples were withdrawn at predetermined time intervals from their respective dissolution vessels to determine the drug released that was immediately replaced with an equal volume of fresh dissolution medium (maintained at 37 ± 0.5 °C) [21]. From initial developmental studies, it was found that during the dissolution of ITZ ASD, a nano-colloidal dispersion is instantly formed, and filtering it directly with a 0.45 μm syringe filter resulted in lower recovery of drug. This could possibly have been due to the absorption/adsorption of drug to the syringe filter [21]. Thus, after systematic analysis, discarding the initial 3 mL of dissolution sample and then using the remaining 2 mL of filtrate for estimation of drug release gave higher drug recovery, which was then applied for all dissolution samples for HPLC analysis [36]. ITZ release was evaluated from each aliquot withdrawn using the Agilent HPLC system 1100 (Agilent Inc., Santa Clara, CA, USA) equipped with a C18 (5 μm, 4.6 × 150 mm) column. A mixture of acetonitrile (75:25 *v*/*v*) and sodium acetate trihydrate aqueous solution (0.04 M) containing triethylamine (0.2% *v*/*v*, pH adjusted to 4.5 using glacial acetic acid) was used as the mobile phase. A flow rate of 1 mL/min and a detection wavelength of 265 nm was used for the analysis of ITZ [21]. The retention time of ITZ was found to be 5.05 ± 0.02 min.

## 3. Results and Discussion

### 3.1. Characterization of Feedstocks

**Figure 3:**
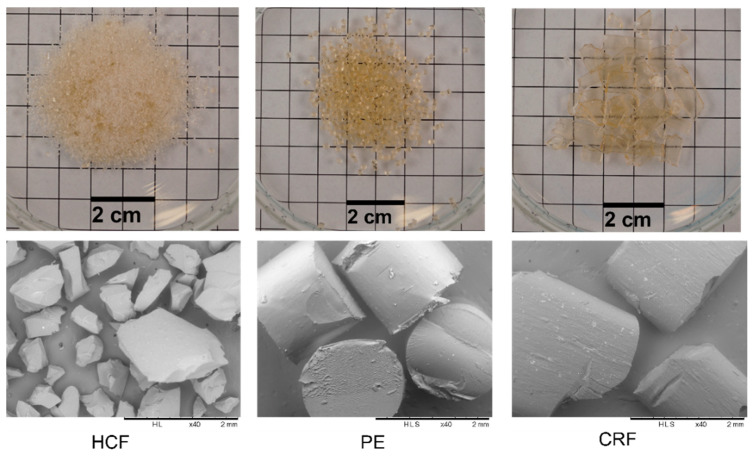
Photograph (**top**) and SEM micrograph (**bottom**) of different feedstocks for milling. From left to right: HCF, PE, CRF.

Change in downstream equipment after the extruder barrel results in differently shaped and sized feedstock materials for milling (Figure 3). The hand-cut filaments of 7–8 cm length were not suitable for direct fine milling and were premilled using a 4 mm screen (Figure 1a). The premilled feedstock (HCF) was the finest material. When using the pelletizer as downstream equipment, regular-shaped cylindrical pellets (PEs) of approximately 2 mm diameter and length were obtained. The chill roll flakes (CRFs) were irregular-shaped shards with different diameters and approximately 1 mm thickness.

To confirm the amorphous state of ITZ after strand processing, the HCF, PE, and CRF feedstocks were analyzed by DSC and pXRD (Figure S3, Supplementary Materials). In the DSC curve, a sharp melting endotherm at 167 °C was found for crystalline ITZ, whereas this was completely absent in the cases of all three feedstocks, thus confirming ITZ in an amorphous state. Similarly, ITZ exhibited several strong characteristic crystalline peaks using pXRD, whereas no crystalline peaks could be observed in the case of the XRD profiles of all three feedstocks, confirming their amorphous nature. The formation of ASD after extrusion of ITZ with HPMCAS has been reported previously [20,21,22,23]. When upscaling the extrusion process using an extruder with a larger screw diameter, it is to be expected that similar feedstock material will be prepared when using a pelletizer or chill roll downstream equipment. In the case of pelletizers, when using an extruder with a larger screw diameter, a die with multiple bores is installed, resulting in multiple strands being fed into the pelletizer. The size and shape of the individual pellets do not change. For the chill roll, when increasing extruder screw diameter, a larger die diameter is used. When the strand with a larger diameter is then subjected to the chill roll, a wider sheet with a similar thickness to the lab-scale process described here is produced. This results in the same thickness of shards after passing through the flake crusher.

### 3.2. Milling of Feedstocks and the Effects of the Independent Variables on the CQAs of the Milled Powders and Tablets

#### 3.2.1. Effect of Independent Variables on Percentage Yield

**Figure 4:**
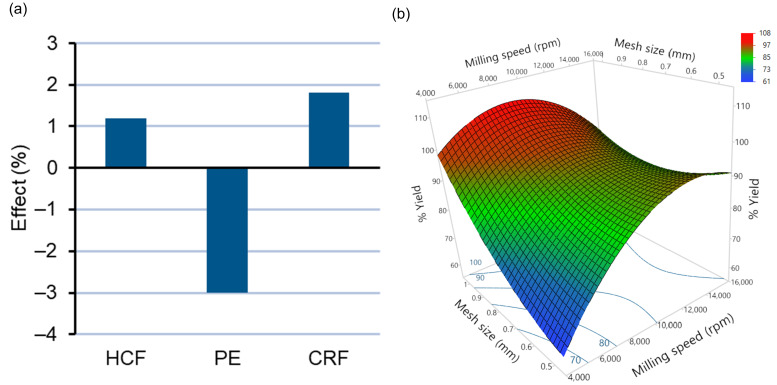
(**a**) Plot depicting the effect of type of feedstock on percentage yield. (**b**) Response surface plot depicting the effects of milling speed and mesh size on percentage yield.

Milling of all three types of feedstocks was carried out with varying milling speeds and mesh diameters and their effects on percentage yield, i.e., output of material after milling, as one of the CQAs, were studied (Table 1). The effects of type of feedstocks (X_1_), milling speed (X_2_), and mesh size (X_3_) have been illustrated with a second-order polynomial equation (Equation (4)). Fitting of the model for percentage yield using customized response surface methodologies was found to be good with a correlation coefficient (R^2^) and an adjusted R^2^ value of 0.9355 and 0.8171, respectively (Table S2, Supplementary Materials). A lower root-mean-square error (RMSE) value of 4.12 also indicated a good correlation between actual and predicted values for percentage yield (Table S2, Supplementary Materials). Statistical analysis with ANOVA indicated the significance according to *t*-testing for the main effects, interactions, and quadratic effects of the DoE terms, tabulated in Table S3 (Supplementary Materials). The effects of type of feedstock and milling speed and mesh size have been illustrated in Figure 4a,b, respectively.

Percentage yield = 91.42 + 1.18X_1_ [HCF] − 3.00X_1_ [PE] + 1.82 X_1_ [CRF] + 6.99X_2_ + 5.30X_3_ − 6.51X_2_^2^ (4)

Via multiple regression analysis, it was found that the percentage yield of ASD extrudates after milling was influenced by the type of feedstock. PE feedstock showed a significant decrease (*p* < 0.05) in percentage yield after 90 s of milling compared to premilled CRF and HCF (Figure 4a). The lower percentage yield in the case of pellets could be attributed to the defined ordered structure of the pellets, due to which breakage of particles occurs along pellet planes, whereas, in the cases of premilled HCF and chill roll flake (CRF) particles, due to their being irregular in shape and size (Figure 3), the efficiency of size reduction is higher due to internal weakness [37]. Similar effects of shape and morphology on degrees of size reduction were observed by Shariare et al. [38], who reported greater size reduction in needle-shaped crystals compared to plate-type crystals of ibuprofen due to their disordered and flawed structure. Among other independent variables, increasing milling speed from 5000 rpm to 15,000 rpm causes significant increase in percentage yield due to the application of higher mechanical energy (Figure 4b) [37]. Similarly, increasing mesh diameter from 0.5 mm to 1 mm causes significant increase in percentage yield due to a decrease in the retention of milled extrudates in the mesh [37,39]. Among other coefficients of the DoE model, only the quadratic effect of milling speed (X_2_^2^) was found to have a significant negative effect (*p* < 0.05) on the percentage yield of milled extrudates, implying a curvature effect of milling speed.

#### 3.2.2. Effect of Independent Variables on Particle Size Distribution

The particle size of extrudates was assessed after milling by laser diffraction. The effects of type of feedstocks (X_1_), milling speed (X_2_), and mesh size (X_3_) have been illustrated using a second-order polynomial equation representing the degree of quantitative effect of each independent variable on D50 (µm) (Equation (5)). The quality of the fit of the model for D50 (μm) was found to be excellent, with R^2^, adjusted R^2^, and RMSE values of 0.9951, 0.98361, and 11.82, respectively (Table S2, Supplementary Materials). The effects of type of feedstock, milling speed, and mesh size, along with statistical analysis by ANOVA, has been summarized in Table S3 (Supplementary Materials).

D50 (µm) = 282.94 + 8.32X_1_ [HCF] + 40.23X_1_ [PE] − 48.56X_1_ [CRF] − 83.92X_2_ + 50.58X_3_ + 20.60X_1_[HCP] × X_2_ − 12.37X_1_ [PE] × X_2_ − 27.990 X_2_ × X_3_ (5)

**Figure 5:**
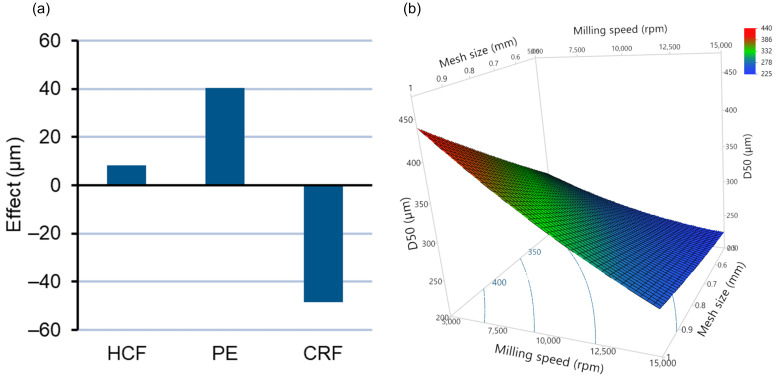
(**a**) Plot depicting the effect of feedstock type on D50 (μm). (**b**) Response surface plot depicting the effects of milling speed and mesh size on D50 (μm).

The effect plot and response surface plot depicting the effects of type of feedstock, milling speed, and mesh size have been illustrated in Figure 5a,b respectively. From the effect plot (Figure 5a) and ANOVA analysis, it can be observed that using chill roll flakes (CRFs) as feedstock, a significant decrease in the mean particle size (D50) of milled extrudates was achieved compared to HCF and PE feedstock. By increasing milling speed from 5000 rpm to 15,000 rpm, a decrease in D50 values occurred due to increased mechanical energy [37], whereas changing the mesh screen from 0.5 to 1.0 mm caused an increase in D50 [39]. As observed from Equation (5), the negative sign for interaction term coefficients of milling speed and mesh size indicates a significant negative effect on D50 values, indicating that simultaneous change of milling speed and mesh size causes a decrease in D50 values.

**Figure 6:**
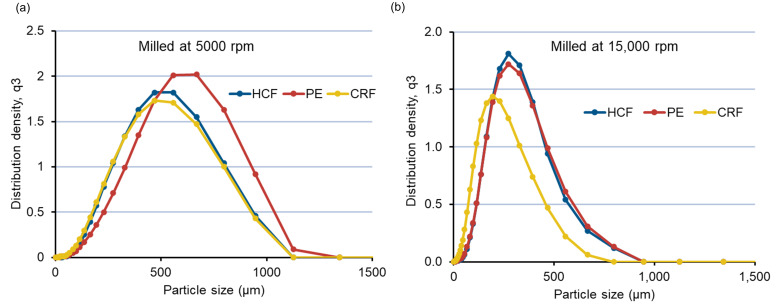
(**a**) PSD curve of feedstocks milled at 5000 rpm using a 1 mm mesh size. (**b**) PSD curve of feedstocks milled at 15,000 rpm using a 1 mm mesh size.

One of the other major reasons for the significant decrease (*p* < 0.05) in mean particle size (D50) observed using CRF feedstock compared to HCF premilled and pellets could be attributed to the higher surface area available for milling due to the reduced thickness of CRF obtained by pressing the molten mixture between two rollers [40]. Additionally, it has also been reported that cooling of a molten mixture at a definite rate improves the overall heat transfer rate, causing inhibition of structural relaxation of the resultant CRF feedstock [40,41]. A lower degree of structural relaxation of extrudates may also cause the increase in the brittleness of material and greater size reduction (lower D50 values) in the case of CRF feedstocks compared to pellets and HCF which can be observed. Further, for better illustration of the effect of feedstocks on PSD, a comparative evaluation of the milling of feedstocks at two different milling speeds (5000 and 15,000 rpm) using similar mesh screens (1.0 mm) was plotted (Figure 6a,b). PSD results for milled feedstocks (D10, D50, and D90) are tabulated in Table 3. As shown in Figure 6a,b, a monomodal size distribution could be observed for the milled ASD extrudates at both 5000 and 15,000 rpm with a 1.0 mm sieve. It was found that at a lower milling speed of 5000 rpm, HCF premilled and CRF feedstock give almost similar PSDs, with D50 values of 436.0 μm and 420.6 μm, respectively (Table 4). At a higher milling speed of 15,000 rpm, PSD is significantly lower for all the feedstocks. However, at a high milling speed, the PE feedstock shows an almost similar PSD compared to the HCF premilled feedstock, with D50 values of 259.7 μm and 259.6 μm, respectively. Thus, with varying milling speed and the same mesh size, a correlation between the PSDs of all three feedstocks (HCF, PE, and CRF) can be established. This is especially important since usually at laboratory scale hand-cut filaments are mostly produced as milling feedstocks for preliminary formulation development studies. A correlation of PSD with other feedstocks, such as pellets and chill roll flakes, will be helpful for the formulation scientist in developing robust processing parameters to achieve the desired PSDs of milled extrudates for industrial production.

Along with PSD, the flow properties as per experimental design have been tabulated in Table S4 (Supplementary Materials). Although the flow properties of milled ASD extrudates were expected to depend largely on PSD, no significant correlation could be established. However, higher bulk and tapped density in the range of 0.500–0.600 g/mL and 0.645–0.769 g/mL was found in 18 experiments with milled extrudates. Using Carr’s index and Hausner’s ratio, the flowability for all the milled extrudates was found to be fair/passable as per USP classifications and thus suitable for direct compression.

#### 3.2.3. Effect of Independent Variables on the Compaction of Milled Granules, Tablet Tensile Strength, and Compaction Analysis

**Figure 7:**
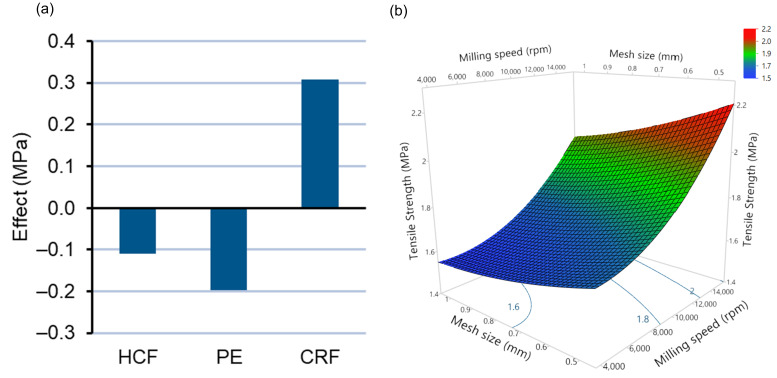
(**a**) Plot depicting the effect of type of feedstock on tensile strength (MPa). (**b**) Response surface plot depicting the effects of milling speed and mesh size on tensile strength (MPa).

The effect of type of feedstocks, milling speed, and mesh size on ITZ:HPMCAS ASD tablet tensile strength with 50% loading has been illustrated by effect plot (Figure 7a) and response surface plot (Figure 7b), respectively. A linear model was found to be significant (*p* < 0.05) in elucidating the effect of the independent variables on tensile strength, with no significant quadratic or interaction effects. The quality of the fit of the model was found to be high, with a correlation coefficient (R^2^) = 0.9395 and a root-mean-square error (RMSE) = 0.15 indicating an excellent correlation between observed and predicted values for tensile strength (Table S2, Supplementary Materials). From multiple regression analysis and ANOVA, it was found that, among the types of feedstocks, CRF caused a significant increase in the tensile strength of tablets (Table S3, Supplementary Materials), whereas, when pellets were used as feedstock, a significant decrease in the tensile strength of tablets occurred (Equation (6)). The effect of HCF feedstock with respect to decrease in tensile strength was found to be insignificant (*p* > 0.05) (Table S3, Supplementary Materials). Among other independent variables, increase in milling speed (X_2_) and decease in mesh size (X_3_) caused significant increases in the tensile strength of tablets due to decrease in particle size (Equation (6)) (Table S3, Supplementary Materials).

Tensile strength (MPa) = 1.82 − 0.10X_1_[HCF] − 0.19X_1_[PE]* + 0.31X_1_[CRF]* + 0.26X_2_ − 0.019X_3_ (6)

The contrary effect of CRF and pellets on the tensile strength of tablets can be attributed to the resultant differences in particle size. As already illustrated and discussed in Figure 5, CRF feedstock produces ASD extrudates of smaller particle size compared to pellets, causing an increase in bonding surface area. As the consolidation of particles largely depends on the bonding area and bonding strength available, the resultant tensile strength of tablets is higher when using CRF as feedstock compared to pellets [42]. To further illustrate the significance of the PSD of milled ASD extrudates, a Pearson correlation coefficient test was conducted to study the relationship between D50 (μm) and tensile strength (MPa) for all 18 tablet formulations as per the experimental design (Figure S4, Supplementary Materials). A significant (*p* < 0.05) negative relationship of r = −0.8506 was found between D50 and tensile strength, indicating that an increase in D50 causes a significant decrease in the tensile strength of ASD tablets.

##### Compaction Analysis

**Figure 8:**
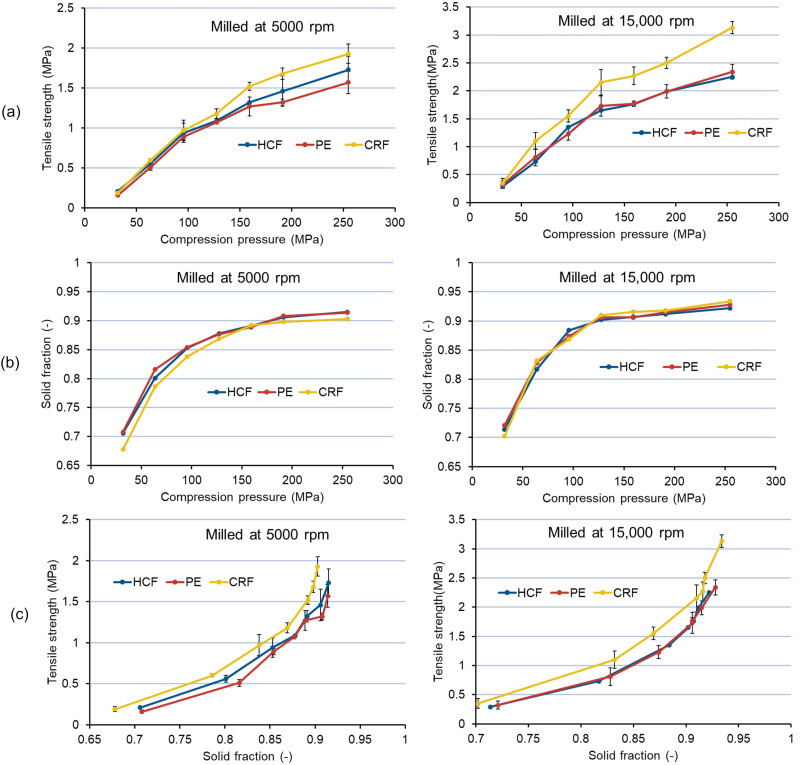
Compaction behavior of milled ASD extrudates: (**a**) tabletability profile, (**b**) compressibility profile, (**c**) compactibility profile. PSD of milled extrudates at both milling speeds (5000 and 15,000 rpm) is summarized in Table 4.

The mechanics of tablet formation can be understood by determining the tabletability, compressibility, and compactibility profiles of powders [33]. To investigate the effect of PSD on compaction behavior, all three feedstocks milled at two different milling speeds (5000 and 15,000 rpm) using the same mesh size (1.0 mm) were compacted into tablets, according to Table 3. From the tabletability profiles (tensile strength vs. compression pressure), it can be observed that CRF feedstock milled at 5000 and 15,000 rpm shows the highest tabletability (Figure 8a). Similarly, from the compactibility profile (tensile strength vs. solid fraction) of milled extrudates (Figure 8c), the highest compactibility at both milling speeds could be observed for CRF feedstock. However, the compressibility profiles (solid fraction vs. compression pressure) at both milling speeds for all three feedstocks were found to be similar (Figure 8b), thus confirming that the compressibility or degree of volume reduction of milled feedstock with the application of compression pressure gives similar results (with HCF, PE, and CRF) [33]. As the work of compaction and deformation/fragmentation is similar in the cases of all three feedstocks, the distinct increase in tabletability and compactibility in the case of CRF can be attributed to the smaller particle size achieved after milling (Table 4) [43], thus confirming the dependence of the compaction and bonding behavior of milled extrudates largely on PSD and the type of sizing technology used. Additionally, we hypothesize that the high cooling rate during CRF manufacturing can inhibit structural relaxation in comparison with HCF and PE feedstocks. This can possibly lead to the lower elasticity and higher compactibility of materials cooled down with a chill roll in comparison to materials cooled by air (HCF and PE) [41].

#### 3.2.4. Effect of Independent Variables on the Dissolution of ITZ:HPMCAS ASD Tablets

**Figure 9:**
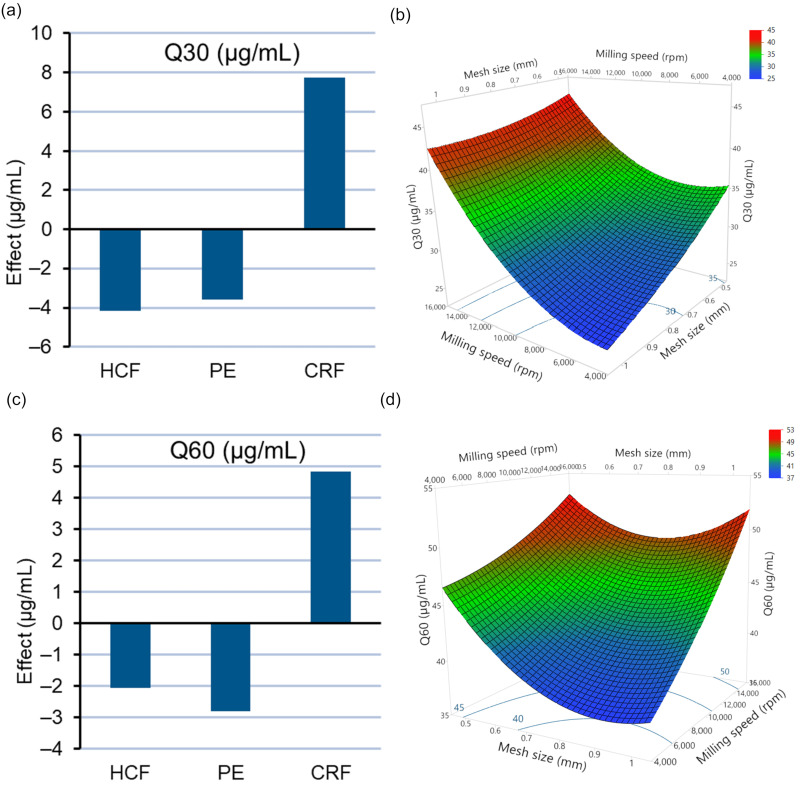
(**a**) Plot depicting the effect of type of feedstock on Q30 (μg/mL). (**b**) Response surface plot depicting the effects of milling speed and mesh size on Q30 (μg/mL). (**c**) Plot depicting the effect of type of feedstock on Q60 (μg/mL). (**d**) Response surface plot depicting the effects of milling speed and mesh size on Q60 (μg/mL).

Rapid disintegration of a tablet into smaller particles is essential for fast release of the drug [8,12]. All tablets prepared in the experimental design disintegrated within one minute (Table S5, Supplementary Materials). The fast disintegration of all ITZ ASD tablet formulations can be attributed to the presence of L-HPC NBD-021 as a disintegrant which has high water absorption and fast swelling action, causing the rapid disintegration of tablets without the formation of gels [12,27]. To assess the dissolution behavior of the compacts, the effect of different feedstocks (X_1_), milling speeds (X_2_), and mesh screens (X_3_) on the release of ITZ (μg/mL) from tablets at 30 min (Q30) and 60 min (Q60) was elucidated using an effect plot (Figure 9a,c) and a response surface plot (Figure 9b,d). The quality of fit of the model for both Q30 and Q60 was found to be excellent, with higher R^2^ values and RMSE values (Table S2, Supplementary Materials). From the polynomial equation results generated by multiple regression analysis (Equations (7) and (8)) and ANOVA (Table S3, Supplementary Materials), it was found that only the main effects of the independent variables have significant effects (*p* < 0.05) on ITZ release, with no effect of quadratic or interaction terms. Among feedstocks, tablets prepared after the milling of CRF feedstock showed significant increases in ITZ release at 30 and 60 min, whereas using HCF and pellet feedstocks was found to have significantly reduced Q30 and Q60 values (Figure 9a,c). With regard to the milling processes, it was found that increasing milling speed and reducing mesh size causes an increase in Q30 and Q60, respectively (Figure 9b,d).

Q30 (µg/mL) = 36.01 − 4.15X_1_[HCF] − 3.59X_1_ [PE] + 7.75X_1_[CRF] + 5.99X_2_ − 3.60X_3_ (7)

Q60 (µg/mL) = 44.73 − 2.05X_1_[HCF] − 2.80X_1_[PE] + 4.85X_1_ [CRF] + 4.68X_2_ − 2.73X_3_ (8)

**Figure 10:**
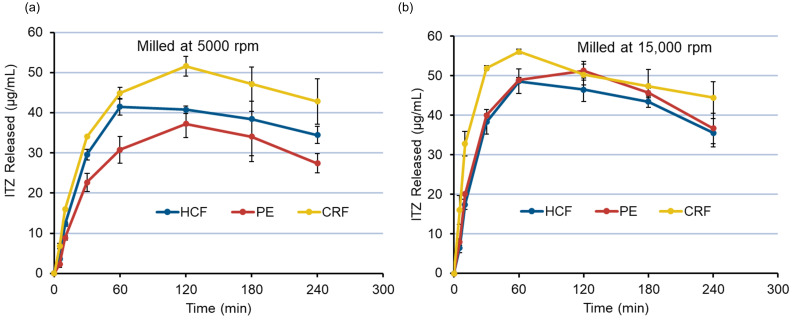
Dissolution of ITZ from ASD tablets using different feedstocks. (**a**) Feedstocks milled at 5000 rpm using a 1 mm mesh screen. (**b**) Feedstocks milled at 15,000 rpm using a 1 mm mesh screen. PSDs of milled extrudates at both milling speeds (5000 and 15,000 rpm) are summarized in Table 4.

The mean Q30 and Q60 values for ITZ ASD tablets from the experimental design were found to be 37.90 μg/mL and 46.35 μg/mL, respectively. The solubility of crystalline ITZ at pH 6.8 is approximately 4 ng/mL (0.004 μg/mL) (15). Thus, an increase in the solubility of ITZ of approximately 9500 times and 12,000 times at 30 and 60 min could be observed for ITZ ASD tablets with HPMCAS as carrier polymer. The ITZ dissolution from ASD tablet is largely related to the resultant PSDs of the milled ASD extrudates. This can be illustrated with the dissolution of ITZ of different feedstocks (HCF, PE, and CRF) milled at two different milling speeds using the same mesh screen (Figure 10). The PSD data for the milled extrudates at 5000 and 15,000 rpm using a 1 mm mesh have already been presented in Table 4. As observed at a milling speed of 5000 rpm (Figure 10a), Q30 (µg/mL) and Q60 (µg/mL) is correlated with the PSDs of feedstocks (Table 4). However, higher supersaturation is achieved by CRF feedstock compared to HCF despite their having almost similar PSDs, whereas, with a higher milling speed of 15,000 rpm (Figure 10b), the Q30 and Q60 values and the supersaturation of ITZ was found to be PSD-dependent, with CRF feedstocks showing the fastest release of ITZ with instant supersaturation compared to HCF and PE. As smaller ASD particles have increased specific surface areas, a higher dissolution of ITZ occurs when using CRF feedstocks compared to HCF and pellets. A similar significant increase in the dissolution of Ketoconazole:HPMCAS ASD with smaller size fractions of ASDs has been reported by Monschke et al. [44]. Additionally, the smaller particle size might also increase the wettability of the drug, thus causing an overall increase in the dissolution of ITZ when CRF is used as feedstock [45]. The importance of PSD in the release of ITZ from ASD tablets was further confirmed by the Pearson correlation coefficient test for the D50 (μm) values of all 18 experiments, with resultant Q30 (μg/mL) and Q60 values (μg/mL) (Figure S5, Supplementary Materials). A significant negative correlation (*p* < 0.05) of −0.8659 and −0.8749 of D50 with Q30 (μg/mL) and Q60 (μg/mL), respectively, was found, indicating that a lower D50 of milled extrudates causes a higher dissolution (higher Q30 and Q60) of ITZ from ASD tablets, thus confirming the significance of PSD for the dissolution of ITZ from ASD tablets.

## 4. Conclusions

Poor aqueous solubility remains one of the major challenges relating to the oral bioavailability of drugs. Although HME is one of the technologies industrially applied to improve the solubility and resultant bioavailability of poorly soluble drugs, the downstream processing of extrudates, including sizing before milling, milling, and tableting, is challenging. In the present study, we established a robust platform for downstream processing of HME extrudates based on an ASD of poorly soluble itraconazole with hypromellose acetate succinate as a carrier polymer. After extrusion, the strands were sized into three types of ITZ ASD feedstocks before milling in the form of hand-cut filaments (premilled), pellets, and chill roll flakes. The effects of feedstock shape, feedstock milling speed, and milling mesh size on granules and tablet CQAs were systematically investigated using response surface methodologies. It was found that, among the feedstocks, the highest percentage yield with a lower PSD was observed for chill roll flakes compared to hand cut filaments and pellets. The tensile strength and ITZ release from ASD tablets were also found to be the highest after the milling of chill roll flake feedstock. A detailed analysis of results using the Pearson correlation coefficient test indicated that the lower PSD achieved in the case of CRF feedstocks resulted in the higher tabletability and dissolution of ITZ, indicating PSD as the most important CQA of milled ASD extrudates. Furthermore, although the feedstock for milling showed morphological differences, it was found that by careful selection of milling speed, a similar PSD was observed after the milling of hand-cut filaments in comparison with the milling of pellets or chill roll flakes, demonstrating an easy transfer from early-formulation hand-cut processing at the lab scale to industrial-applicable processes. Thus, a comprehensive understanding of the effect of different feedstocks and milling process parameters on the downstream processing of Itraconazole ASD into tablets with high tensile strength and fast disintegration and dissolution was achieved in the present study.

## Figures and Tables

**Table 1:** Experimental design for studying the effect of independent variables on CQAs of ITZ:HPMCAS ASD extrudates.

Independent Variables			
	Type I	Type 2	Type 3
Type of Feedstock ^a^	HCF	PE	CRF
	Low	Medium	High
Milling speed (rpm) ^b^	5000	10,000	15,000
Mesh size (mm) ^b^	0.5	0.75	1
**Responses (CQAs)**			
Milled granule yield (%)			
Milled granule D50 (volume mean particle size in µm)			
Tablet tensile strength (MPa)			
Tablet dissolution Q30 (ITZ release at 30 min)			
Tablet dissolution Q60 (ITZ release at 60 min)			

^a^ Type of feedstock was defined as a categorical variable. ^b^ Milling speed and mesh size were defined as continuous variables.

**Table 2:** Experimental runs as per the experimental design.

Exp. No.	Type of Feedstock	Milling Speed (rpm)	Mesh Diameter (mm)
1	HCF	5000	0.5
2	PE	15,000	0.75
3	PE	5000	0.75
4	CRF	15,000	1
5	HCF	5000	1
6	CRF	15,000	0.5
7	CRF	10,000	0.75
8	HCF	10,000	0.75
9	HCF	15,000	1
10	HCF	15,000	0.5
11	PE	5000	1
12	HCF	10,000	0.75
13	CRF	10,000	0.75
14	CRF	5000	0.5
15	CRF	5000	1
16	PE	15,000	1
17	PE	10,000	1
18	PE	10,000	0.5

**Table 3:** Tablet formulation.

Material	Functionality	*w*/*w* (%)	mg/Tablet (mg)
ITZ:HPMCAS 20:80 ASD	ASD	50.0	325
L-HPC NBD-021	Binder/disintegrant [8,27]	7.5	49
MCC PH 102	Diluent	41.5	270
Silicon dioxide	Glidant	0.5	3
Mg Stearate	Lubricant	0.5	3

**Table 4:** Particle size distribution of the ITZ ASD feedstocks milled at 5000 rpm and 15,000 rpm using a 1 mm mesh screen.

	Milled at 5000 rpm, 1 mm Mesh			Milled at 15,000 rpm, 1 mm Mesh		
Type of Feedstock	D10 (µm)	D50 (µm)	D90 (µm)	D10 (µm)	D50 (µm)	D90 (µm)
HCF premilled	197.59	436.02	753.22	162.49	259.72	501.2
PE	227.53	517.48	840.90	118.69	259.65	491.75
CRF	177.3	420.69	743.83	70.03	178.68	376.58

## Supplementary Materials

The following supplementary materials can be downloaded at: https://www.mdpi.com/article/10.3390/pharmaceutics14071429/s1. Figure S1: Screw design for hot melt extrusion of ITZ:HPMCAS AS-MMP (20:80); Figure S2: Tablet tensile strength vs. ASD load in the tablet. HCF was used as feedstock for tablet compaction as per Table 3 in main document; Figure S3: Solid state characterization of Itraconazole, HPMCAS AS-MMP and ITZ:HPMCAS ASD feedstocks (HCF, PE and CRF). (a) DSC (b) pXRD; FigureS4: Pearson correlation coefficient relationship between D50 (μm) with tensile strength (MPa); FigureS5: Pearson correlation coefficient relationship between D50 (μm) with Q30 (μg/mL) (left), and D50 (µm) with Q60 (µg/mL) (right); Table S1: Preliminary formulation trails to evaluate optimum drug load; Table S2: Summary of fit for response variables according to the experimental design, Table S3: Statistical analysis of effect of independent variable on CQAs of ITZ ASD extrudates by ANOVA. *p* < 0.05 is significant; Table S4: Flow properties of milled ASD extrudates of as per experimental design; Table S5: Disintegration time of ITZ ASD tablet formulation prepared as per experimental design.
