# Supplementary material for: Downstream Processing of Itraconazole:HPMCAS Amorphous Solid Dispersion: From Hot-Melt Extrudate to Tablet Using a Quality by Design Approach
Source: Pharmaceutics. 2022 Jul 7;14(7):1429. doi: 10.3390/pharmaceutics14071429 (PMC9323274; doi:10.3390/pharmaceutics14071429)
Supplement: Supplementary file 1 [file pharmaceutics-14-01429-s001.zip › pharmaceutics-1773654-supplementary.pdf]

Downstream Processing of Itraconazole:HPMCAS Amorphous Solid Dispersion: From Hot-Melt Extrudate to Tablet using a Quality by Design Approach

Authors: Saurabh M Mishra[1], Margarethe Richter[2], Luis Mejia[1] and Andreas Sauer[3,*]

Affiliations: [1] SE Tylose USA Inc. Pharmaceutical Application Laboratory, Totowa, New Jersey-07512, USA

[2] Thermo Fisher Scientific, Pfannkuchstrasse 10-12, 76185 Karlsruhe, Germany

[3] SE Tylose GmbH & Co. KG, Kasteler Str. 45, 65203 Wiesbaden, Germany

Corresponding Author:

Dr. Andreas Sauer

SE Tylose GmbH & Co. KG, Kasteler Str. 45, 65203 Wiesbaden, Germany

Tel: +496119626345 Email: andreas.sauer@setylose.com

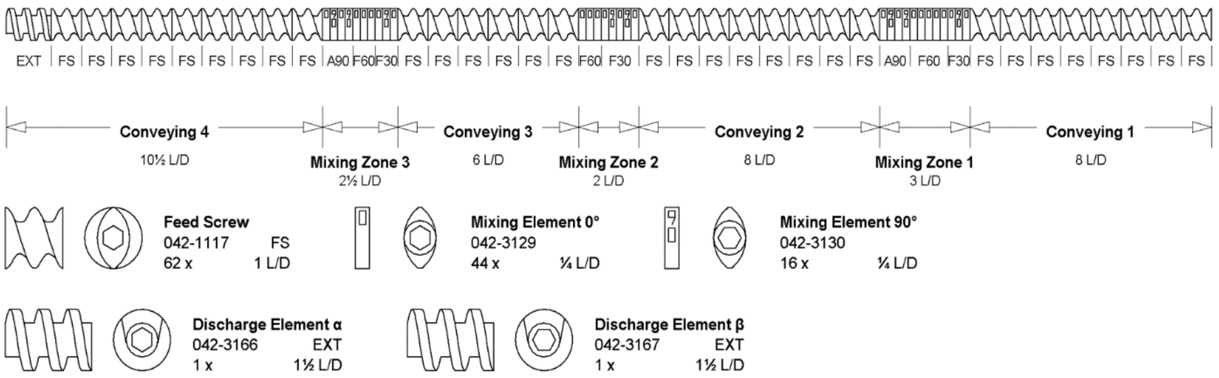

Figure S1. Screw design for hot melt extrusion of ITZ:HPMCAS AS-MMP (20:80).

Table S1. Preliminary formulation trails to evaluate optimum drug load.

Ingredient	**Functionality**	F-1(%)	F-2 (%)	F-3 (%)
ITZ:AS-MMP (20:80) milled extrudates	ASD	33.3	50	67
NBD-021	Binder/Disintegrant	7.5	7.5	7.5
MCC PH 102	Diluent	58.2	41.5	24.5
Silicon dioxide	Glidant	0.5	0.5	0.5
Mg Stearate	Lubricant	0.5	0.5	0.5
Total	-	100.0	100.0	100.0

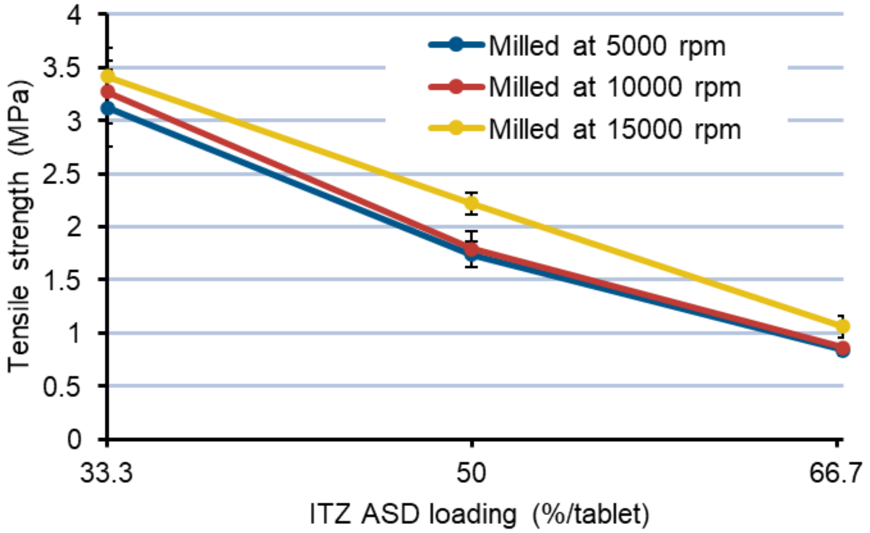

Figure S2. Tablet tensile strength vs. ASD load in the tablet. HCF was used as feedstock for tablet compaction as per Table 3 in main document.

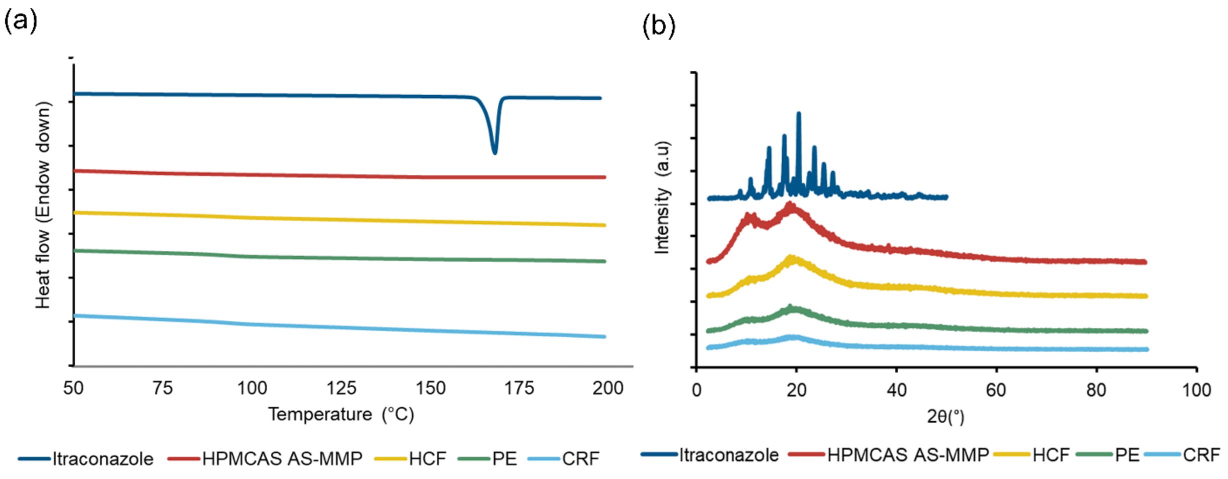

Figure S3. Solid state characterization of Itraconazole, HPMCAS AS-MMP and ITZ:HPMCAS ASD feedstocks (HCF, PE and CRF). (a) DSC (b) pXRD.

Table S2. Summary of fit for response variables according to the experimental design.

Fitting parameters	% Yield	D50 (µm)	Tensile strength (MPa)	ITZ release	
				Q30 (µg/mL)	Q60 (µg/mL)
R^2	0.9355	0.9951	0.9395	0.9314	0.9293
Adj. R^2	0.8171	0.9861	0.8287	0.8056	0.7997
Root Mean Square Error (RMSE)	4.12	11.82	0.15	3.97	3.09
Mean of Response	88.34	297.93	1.89	37.90	46.35
Observations (or Sum Wgts)	18	18	18	18	18

Table S3. Statistical analysis of effect of independent variable on CQAs of ITZ ASD extrudates by ANOVA. P<0.05 is significant.

Term	%yield		D50 (µm)		Tensile strength (MPa)		Q30 (µg/mL)		Q60 (µg/mL)	
	t Ratio	Prob>|t|	t Ratio	Prob>|t|	t Ratio	Prob>|t|	t Ratio	Prob>|t|	t Ratio	Prob>|t|
Intercept	49.01	<.0001	52.99	<.0001	27.65	<.0001	20.06	<.0001	32.01	<.0001
Feedstock [HCF]	0.85	0.4291	2.08	0.0831	-2.21	0.069	-3.08	0.0216	-1.96	0.0982
Feedstock [PE]	-2.04	0.0868	9.57	<.0001	-3.79	0.009	-2.54	0.0439	-2.55	0.0437
Feedstock [CRF]	1.3	0.2419	-12.12	<.0001	6.19	0.0008	5.75	0.0012	4.63	0.0036
Milling speed (rpm)	5.76	0.0012	-24.15	<.0001	6.01	0.001	5.13	0.0021	5.16	0.0021
Milling speed (rpm)*Milling speed (rpm)	-4.01	0.007	1.55	0.1728	1	0.3573	1.28	0.2488	0.4	0.703
Mesh size (mm)	4.27	0.0053	14.23	<.0001	-2.78	0.032	-3.02	0.0235	-2.94	0.0261
Mesh size (mm)*Mesh size (mm)	1.84	0.1153	0.34	0.7463	0.23	0.8247	0.3	0.7759	1.4	0.2104
Feedstock [HCF]*Milling speed (rpm)	-1.41	0.2085	4.23	0.0055	-0.93	0.3883	-0.22	0.8329	-0.39	0.709
Feedstock [PE]*Milling speed (rpm)	1.15	0.2932	-2.47	0.0482	0.36	0.7339	0.66	0.5349	1.91	0.1041
Feedstock [CRF]*Milling speed (rpm)	0.23	0.8287	-1.69	0.1421	0.56	0.5931	-0.46	0.6648	-1.57	0.1665
Feedstock [HCF]*Mesh size (mm)	1.4	0.2117	-1.28	0.248	0.65	0.5381	0.57	0.5893	0.61	0.5621
Feedstock [PE]*Mesh size (mm)	-1.01	0.3513	1.22	0.2687	0.94	0.3856	-0.72	0.5002	-1.56	0.1692
Feedstock [CRF]*Mesh size (mm)	-0.33	0.7547	-0.01	0.9909	-1.64	0.1513	0.19	0.8557	1.04	0.3376
Milling speed (rpm)*Mesh size (mm)	-4.73	0.0032	-7.1	0.0004	-0.33	0.7522	0.97	0.3713	1.27	0.2518

Table S4. Flow properties of milled ASD extrudates of as per experimental design.

Experimental number	Bulk density (g/mL)	Tapped density (g/mL)	Carr's Index	Hausner's ratio	Scale of flowability [27]
1	0.588	0.714	17.65	1.21	Fair
2	0.556	0.740	24.86	1.33	Passable
3	0.571	0.714	20.03	1.25	Fair
4	0.513	0.645	20.47	1.26	Passable
5	0.588	0.769	23.54	1.31	Passable
6	0.513	0.666	22.97	1.30	Passable
7	0.555	0.714	22.27	1.29	Passable
8	0.555	0.714	22.27	1.29	Passable
9	0.588	0.714	17.65	1.21	Fair
10	0.588	0.714	17.65	1.21	Fair
11	0.606	0.741	18.22	1.22	Fair
12	0.588	0.714	17.65	1.21	Fair
13	0.526	0.666	21.02	1.27	Passable
14	0.500	0.645	22.48	1.29	Passable
15	0.526	0.666	21.02	1.27	Passable
16	0.571	0.714	20.03	1.25	Fair
17	0.588	0.714	17.65	1.21	Fair
18	0.588	0.714	17.65	1.21	Fair

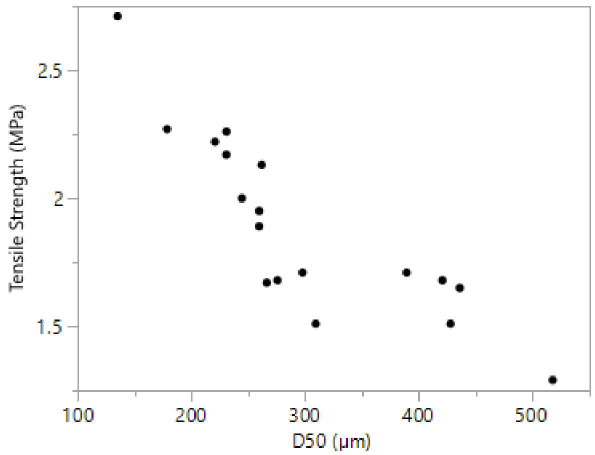

Summary Statistics

	Value	Lower 95%	Upper 95%	Signif. Prob
Correlation	-0.85064	-0.943	-0.63657	<.0001*
Covariance	-30.2191			
Count	18			

Variable	Mean	Std Dev
D50 (μm)	297.9361	100.5496
Tensile Strength (MPa)	1.889444	0.353312

Figure S4. Pearson correlation coefficient relationship between D50 (μm) with tensile strength (MPa).

Table S5. Disintegration time of ITZ ASD tablet formulation prepared as per experimental design.

Exp. No	Feed stocks	Milling speed (rpm)	Mesh size (mm)	Disintegration time (s)
1	HCF	5000	0.5	45 ± 5
2	PE	15000	0.75	60 ± 7
3	PE	5000	0.75	51 ± 9
4	CRF	15000	1	47 ± 6
5	HCF	5000	1	35 ± 4
6	CRF	15000	0.5	62 ± 6
7	CRF	10000	0.75	48 ± 3
8	HCF	10000	0.75	39 ± 4
9	HCF	15000	1	44 ± 4
10	HCF	15000	0.5	49 ± 5
11	PE	5000	1	42 ± 5
12	HCF	10000	0.75	39 ± 3
13	CRF	10000	0.75	50 ± 3
14	CRF	5000	0.5	57 ± 15
15	CRF	5000	1	42 ± 5
16	PE	15000	1	54 ± 3
17	PE	10000	1	46 ± 7
18	PE	10000	0.5	66 ± 5

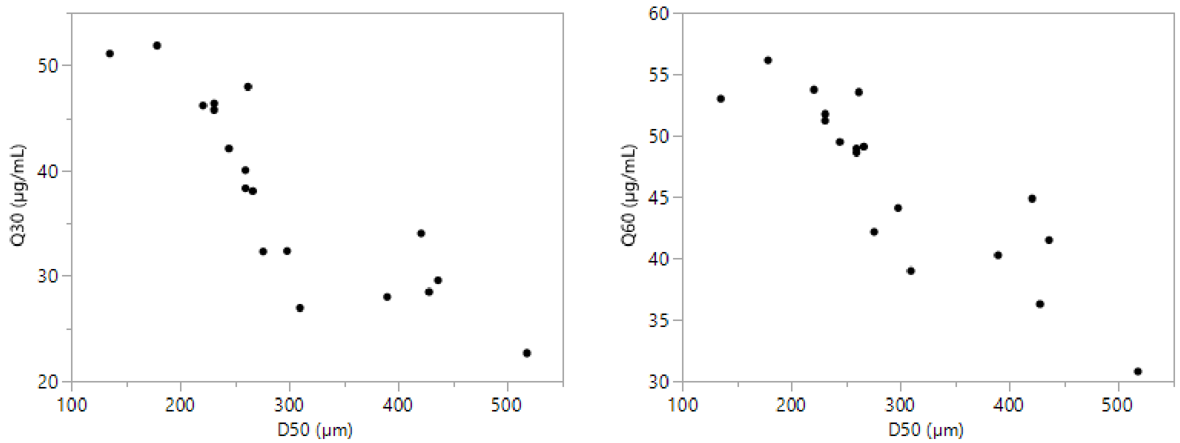

Summary Statistics

	Value	Lower 95%	Upper 95%	Signif. Prob
Correlation	-0.86591	-0.94909	-0.66984	<.0001*
Covariance	-784.786			
Count	18			

Variable	Mean	Std Dev
D50 (µm)	297.9361	100.5496
Q30 (µg/mL)	37.89667	9.013562

Summary Statistics

	Value	Lower 95%	Upper 95%	Signif. Prob
Correlation	-0.87492	-0.95266	-0.68983	<.0001*
Covariance	-608.132			
Count	18			

Variable	Mean	Std Dev
D50 (µm)	297.9361	100.5496
Q60 (µg/mL)	46.34944	6.912713

Figure S5. Pearson correlation coefficient relationship between D50 (µm) with Q30 (µg/mL) (left), and D50 (µm) with Q60 (µg/mL) (right).
